# Issues for the management of people with diabetes and COVID-19 in ICU

**DOI:** 10.1186/s12933-020-01089-2

**Published:** 2020-07-20

**Authors:** Antonio Ceriello, Eberhard Standl, Doina Catrinoiu, Baruch Itzhak, Nebojsa M. Lalic, Dario Rahelic, Oliver Schnell, Jan Škrha, Paul Valensi, the “Diabetes and Cardiovascular Disease (D&CVD)” Study Group of the European Association for the Study of Diabetes (EASD)

**Affiliations:** 1 IRCCS MultiMedica, Via Gaudenzio Fantoli, 16/15, 20138 Milan, Italy; 2 Forschergruppe Diabetes e.V. at Munich Helmholtz Centre, Munich, Germany; 3 Clinical Center of Diabetes, Nutrition and Metabolic Diseases, Faculty of Medicine, Ovidius University of Constanta, Constanta, Romania; 4 Clalit Health Services and Technion Faculty of Medicine, Haifa, Israel; 5 Clinic for Endocrinology, Diabetes and Metabolic Diseases, Faculty of Medicine, University of Belgrade, Belgrade, Serbia; 6 Vuk Vrhovac University Clinic for Diabetes, Endocrinology and Metabolic Diseases, Merkur University Hospital, Zagreb, Croatia; 7 University of Zagreb School of Medicine, Zagreb, Croatia; 8 University of Osijek School of Medicine, Osijek, Croatia; 9 Department of Internal Medicine 3, 1st Faculty of Medicine, Charles University, Prague, Czech Republic; 10 Unit of Endocrinology, Diabetology, Nutrition, Jean Verdier Hospital, APHP, Paris Nord University, Sorbonne Paris Cité, CINFO, CRNH-IdF, Bondy, France

**Keywords:** Cardiovascular complications, COVID-19, Diabetes, Intensive Care Unit

## Abstract

In the pandemic “Corona Virus Disease 2019” (COVID-19) people with diabetes have a high risk to require ICU admission. The management of diabetes in Intensive Care Unit is always challenging, however, when diabetes is present in COVID-19 the situation seems even more complicated. An optimal glycemic control, avoiding acute hyperglycemia, hypoglycemia and glycemic variability may significantly improve the outcome. In this case, intravenous insulin infusion with continuous glucose monitoring should be the choice. No evidence suggests stopping angiotensin-converting-enzyme inhibitors, angiotensin-renin-blockers or statins, even it has been suggested that they may increase the expression of Angiotensin-Converting-Enzyme-2 (ACE2) receptor, which is used by “Severe acute respiratory syndrome coronavirus 2 (SARS-CoV-2) to penetrate into the cells. A real issue is the usefulness of several biomarkers, which have been suggested to be measured during the COVID-19. N-Terminal-pro-Brain Natriuretic-Peptide, D-dimer and hs-Troponin are often increased in diabetes. Their meaning in the case of diabetes and COVID-19 should be therefore very carefully evaluated. Even though we understand that in such a critical situation some of these requests are not so easy to implement, we believe that the best possible action to prevent a worse outcome is essential in any medical act.

## Background

In the pandemic “Corona Virus Disease 2019” (COVID-19) people with diabetes have a high risk to require ICU admission. The management of diabetes in Intensive Care Unit is always challenging, however, when diabetes is present in COVID-19 the situation seems even more complicated. This article discusses the specific problems of managing people with diabetes and COVID-19 in ICU.

In the recent Corona Virus Disease 2019 (COVID-19) pandemic people with diabetes are paying a very high price.

Probably they are not exposed to higher risk of being infected, however, in the case, particularly when the metabolic control is not sufficient, they are more prone to serious complications and to die [1–5]. The rates of severe disease are significantly higher in patients with diabetes compared with non-diabetes (34.6% vs. 14.2) [5]. Similarly, type 2 diabetic patients have higher rates of need for Intensive Care Unit (ICU), (37.0% vs. 26.7%) [5].

It is well recognized that the management of people with diabetes in an ICU is particularly challenging [6]. To this situation we have to add that diabetes is very often accompanied by co-morbidities, such as cardiovascular disease, hypertension and obesity, which by themselves worsen the prognosis of people with COVID-19 [1–5]. Moreover, there are also several other conditions (described in the course of the article), commonly present in diabetes, which can expose people with diabetes and COVID-19 at high risk for complications. It seems, therefore, quite clear that people with diabetes may have a particular profile/needs when hospitalized in ICU for the COVID-19. In this article we seek to discuss the specific issues to which people with diabetes can be exposed in ICU when having the COVID-19.

## Glycemic control

It is well recognized that an optimal glycemic control during the stay in ICU can improve the prognosis [7]. However, the optimal glycemic control, particularly in ICU involves today several aspects. Unfortunately, it is not surprising that patients suffering from COVID-19 with hyperglycemia may have a higher risk and a poorer outcome compared with those with euglycemia [8]. In particular, very recent reports from the USA have shown that uncontrolled glycemia is exposing people with diabetes and COVID-19 at a very high risk to develop serious complications or to die [9, 10].

Evidence shows that in ICU the more time the patients spend in the normal range of glycemia, the better is their prognosis [11, 12]. This is also the case of COVID-19. It has been reported that a tight glycemic control with insulin infusion had a lower risk of severe disease than patients without insulin infusion [13].

This aspect might be of immediate understanding, however, it also implies to recognize why acute peaks of glycemia, episodes of hypoglycemia or, even worse, the exposure of the patients to huge glucose variability during the stay in ICU are all rather deleterious. Acute hyperglycemia produces oxidative stress followed by an enormous production of inflammatory cytokines [14], a situation that obviously must be avoided during any stay in ICU, but particularly during the stay for COVID-19. It is well known that during this disease a massive cytokines storm can occur, with severely damaging effects [15]. Hypoglycemia can produce the same effects as acute hyperglycemia and can expose directly people to the risk of dying [16, 17]. Furthermore, how hypoglycemia is recovered might be dangerous: hyperglycemia post-hypoglycemia is also an issue, leading to an enhancement of inflammation [18].

Finally, there are plenty of reports in the literature that glucose variability is producing a worsening of the prognosis in ICU [16, 19–22] even when glucose is kept in normal range [22]. So it seems advisable that glucose variability should be avoided. Glucose variability also induces the generation of the oxidative stress and the release of inflammatory cytokines [23].

Is the issue of glycemic control also of importance for people with diabetes and COVID-19? It seems, unfortunately, the case, according to reports on how glycemia was managed in several situations during this pandemic [24, 25]. When facing high glucose levels due to severe infection per se, it is often required that patients are switched to insulin, with some concerns that insulin treatment might not always be safely managed in such situations, unless insulin is administered intravenously via an exactly dosing perfusion device to avoid subcutaneous absorption irregularities in critically ill patients [26, 27]. Hyperglycemia is common in the Intensive Care Unit (ICU) both in patients with and without a previous diagnosis of diabetes [28]. The optimal glucose range in the ICU population is still a matter of debate. Given the risk of hypoglycemia associated with intensive insulin therapy, current recommendations include treating hyperglycemia after two consecutive glucose > 180 mg/dL with target levels of 140–180 mg/dL for most patients [28]. The optimal method of sampling glucose and delivery of insulin in critically ill patients remains elusive. While point of care glucose meters are not consistently accurate and have to be used with caution, continuous glucose monitoring (CGM) is not standard of care and is not yet generally recommended for inpatient use. The advent of new technologies, such as electronic glucose management, CGM, and closed-loop systems, promises to improve inpatient glycemic control in the critically ill with lower rates of hypoglycemia [28].

The issue of optimal glycemic control is certainly even more complicated during the management of COVID-19 because high doses of glucocorticoids are often used [29].

Glucocorticoids improve the prognosis of COVID-19 but, of course, induce or worsen hyperglycemia [30]. In the case keeping normal glycemia may be very challenging [30].

**Fig. 1:**
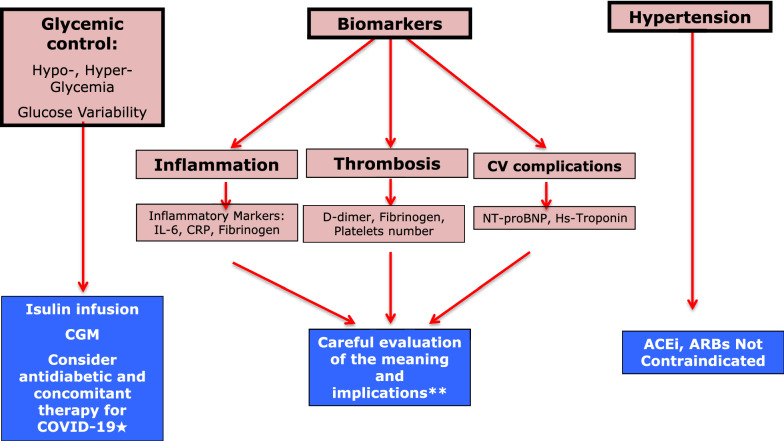
Possible issues in the management of people with diabetes and COVID-19 in ICU. Several issues are present during the management of people with diabetes and COVID-19 in ICU. Tight glycemic control, avoiding hypoglycemia and glucose variability improves the prognosis. This goal, to be achieved, needs insulin infusion and continuous glucose monitoring (CGM). *Moreover, the achievement of tight glucose control may be influenced by the background anti-hyperglycemic therapy and by the concomitant therapy with hydroxychloroquine (risk of hypoglycemia) or corticosteroids (inducing hyperglycemia). **The evaluation of the meaning of several biomarkers related to the risk of cardiovascular complications, thrombosis and inflammation must be careful, because many of them are already altered by diabetes itself. There is no contraindication, however, to the use of ACEi or ARBS to control blood pressure. *ACEi* angiotensin-converting-enzyme inhibitors, *ARBs* angiotensin-receptor-blockers, *CV* cardio-vascular, *CGM* continuous glucose monitoring, *CRP* C-reactive protein, *Hs*-*Troponin* High-sensitive troponin, *ICU* Intensive Care Unit, *IL*-*6* interleukin-6, *NT*-*proBNP* N-Terminal-pro-Brain Natriuretic-Peptide

Another challenge in managing glycemia during the stay in ICU, particularly during the early phase, is the background anti-hyperglycemia therapy. While for several therapies, such as dipeptidyl-peptidase 4 inhibitors (DPP4 inhibitors), sodium-glucose-transporter-2 inhibitors (SGLT-2 inhibitors), pioglitazone, alpha-glucosidase inhibitors, metformin and short-acting Glucagon-Like-Peptide-1 Receptor Agonists (GLP-1RA) (exenatide and lixisenatide) their action is only shortly enduring after they are stopped, this cannot be the case for long-acting insulins but particularly for the long-acting GLP-1RA (dulaglutide, exenatide LA, liraglutide and semaglutide) [31]. Their action will add to that of insulin used during the treatment in ICU and must be considered in choosing the insulin dose. On the other hand, many of them show an anti-inflammatory activity, which could be quite helpful during COVID-19 [32] (Fig. 1).

Another rising problem might be the concomitant use of hydroxychloroquine that, even it is controversial [33], sometimes is used for preventing the effect of the “Severe acute respiratory syndrome coronavirus 2” (SARS-CoV-2) [34]. Hydroxychloroquine has a proven hypoglycemic effect, therefore also in this case the insulin treatment must be very carefully managed in order to avoid episodes of hypoglycemia [35]. Intriguingly, there is also evidence that optimal Covid-19 infection management with Tocilizumab is not achieved during hyperglycemia in both diabetic and non-diabetic patients [36].

Increased attention is needed regarding the proper hydration of the diabetic patient with COVID-19 in the ICU [37]. Hyperhydration can induce the onset of life-threatening pulmonary oedema due to the severity of lung damage during corona infection. Serum K levels are equally important, with a major risk of hypokalaemia, frequently associated with COVID-19, possibly due to hyperaldosteronism caused by elevated angiotensin 2 [37]. Insulin treatment may worsen hypokalaemia if not corrected in time [38].

## Hypertension

Diabetes is very often accompanied by hypertension [39]. Fortunately, the issue of the possible role of angiotensin-converting-enzyme inhibitors and angiotensin-receptor-blockers in favoring the penetration of the SARS-CoV-2, due to the increasing receptor for the virus “Angiotensin-Converting-Enzyme-2” (ACE2), seems to be over [40]. New specific data coming from people with the COVID-19, are certainly reassuring on this point [41, 42]. In the CORONADO study, which included 1317 patients with diabetes, neither hypertension nor treatment by renin–angiotensin–aldosterone system blockers were associated with a worse prognosis [43]. Anyhow, the control of blood pressure remains an important point in the management of people in ICU and of course this particularly applies to people with diabetes.

## The issue of the evaluation of biomarkers meaning in diabetes

Several biomarkers have been suggested to be helpful in stratification of the risk during the COVID-19. Their value as helpful tools in the case of COVID-19 in people with diabetes needs a careful evaluation.

### Biomarkers of cardiovascular disease and heart failure

COVID-19 can cause serious acute cardiovascular events [44]. Moreover, people with a previous cardiovascular disease are more prone to a worse prognosis if affected by SARS-CoV-2 [1–5]. A pre-existing cardiovascular disease very often accompanies diabetes, therefore, people with diabetes also for this reason may be exposed to a more serious complication when having the COVID-19. Furthermore, a large proportion of people with diabetes has asymptomatic coronary artery disease [45, 46] which can increase the risk of acute coronary syndrome, heart failure and arrhythmia during the COVID-19 due to proinflammatory process, hypercoagulability and sympathetic stimulation. QT interval is also often increased in people with diabetes, as a consequence of cardiac autonomic neuropathy [47, 48]. QT interval may be further lengthened by hypoglycemia and by drugs used during the COVID-19 and by hypokalemia and needs to be carefully monitored.

Hs-troponin has been suggested for monitoring the risk of myocardial infarction during the COVID-19 [49, 50]. However, in the case of diabetes, increased levels of hs-troponin have been reported [47], as signal of an existing chronic heart damage, therefore its use for monitoring heart risk in diabetes during COVID-19 deserves some caution.

The situation is not different for heart failure, which again, has been described as a serious complication of COVID-19 [49, 50]. The measurement of the “N-Terminal-pro-Brain Natriuretic-Peptide” (NT-proBNP) to define the risk for heart failure in COVID-19 has been suggested [48–50]. However, plasma NT-proBNP level in a patient with COVID-19 must be seen as a marker of both the presence and extent of pre-existing cardiac disease and the acute haemodynamic stress related to COVID-19. As for hs-troponin, increased levels of NT-proBNP have been often reported in diabetes [51, 52]. According to various studies, an asymptomatic heart failure can be present in up to more than 50% of people with diabetes [51–53]. This condition is characterized by an increase of plasma NT-proBNP. So, again, the usefulness of a marker, in this case the NT-proBNP, needs a careful evaluation in the presence of diabetes and COVID-19.

### Thrombosis biomarkers

Thrombosis has been found to occur very often during the COVID-19 and it is one of the most serious complications [54]. The measurement of D-dimer is very useful in the prediction of the risk for a thrombotic event and its evaluation has been suggested regarding the management of COVID-19 patients [55, 56].

The situation in diabetes looks more complicated. D-dimer is often elevated in diabetes [57, 58]. It is just the index of increased thrombophilia, which is highly prevalent in diabetes [59]. The thrombophilia in diabetes is related to an imbalance between thrombosis and fibrinolysis [59]. This status suggests in one side that the evaluation of the thrombotic status in diabetes during the admission in ICU using the D-dimer should be very careful, but at the same time that an anticoagulation could be very helpful.

In this context the role of hyperglycemia also deserves attention. An acute increase of glycemia may activate thrombin formation [60], convincingly through the glycation of the antithrombin III, a phenomenon that can be reversed by a fast control of hyperglycemia or by heparin [61]. Heparin administration is largely suggested in the case of COVID-19, so it seems true that there is further reason for its use in the presence of diabetes [50]. Finally, it has been recently reported that glucose variability may increase the platelet reactivity [62]. Therefore, regarding the risk of thrombosis, there are good reasons to keep glycemia under a strict control, in association with anticoagulation.

### Inflammation biomarkers

Markers of inflammation, particularly C-reactive-protein and interleukin-6 have also been suggested as tools for monitoring the severity of the COVID-19 [50, 55]. Again diabetes has a particular situation. Low-grade inflammation is present in this disease [63], therefore, as for thrombosis and heart failure, the significance of altered values of these markers needs a careful evaluation in people with diabetes and COVID-19. Furthermore, as reported above, an acute increase of glycemia as well as glucose variability are accompanied by an increased production of cytokines [64, 65], an effect that must be avoided in the COVID-19.

## Lipids and statin use

Most of diabetic patients are routinely on lipid-lowering treatment, in particular on statins in accordance with the current diabetes and cardiovascular disease guidelines [66]. Statins have well-known anti-inflammatory effects and improve endothelial function, which may be protective against cardiovascular complications during COVID-19. However, through various mechanisms statins may enhance compensatory immune signals [67]. In addition, similar to renin–angiotensin–aldosterone system blockers, experimental studies showed that statins also augment the ACE2 receptor expression [68] and might thus facilitate the penetration of SARS-CoV-2 into the cells. Whether statins may be beneficial or harmful during virus-induced acute respiratory distress syndrome is controversial [69, 70]. Further investigations are urgently required to clarify the interplay of these complex mechanisms with the new coronaviruses. In addition statins may cause myotoxicity. A markedly increase in creatine kinase may be observed in some patients with COVID-19 [1].

The benefit of statins in cardiovascular prevention is well established in people with diabetes. In this context there is no evidence for withdrawing statins during COVID-19. However creatin kinase should be carefully monitored and, if increased, statin therapy should be temporarily withheld in order to avoid rhabdomyolysis.

## Type 1 diabetes

Probably it is not well recognized that all we have reported above, which is true for type 2 diabetes, applies also and particularly and more seriously to type 1 diabetes [71, 72]. Type 1 diabetes has the same, albeit not more than type 2 diabetes, risk for cardiovascular events [71]. Moreover, it is important to note that many old people may have today type 1 diabetes. Data are reassuring, showing that people with type 1 diabetes are not more exposed to SARS-COV-2 infection [73] nor to more severe outcomes compared to patients with type 2 diabetes [43]. However, type 1 diabetes is more complicated to manage and probably deserves a special attention when admitted to ICU for COVID-19.

## Conclusions

Managing people with diabetes in any acute setting is always very difficult. COVID-19 has a very severe prognosis for people with diabetes and evidence shows that a tight glucose control could be very helpful. In the case of COVID-19 people with diabetes are more exposed to cardiovascular complications, which may be more challenging to manage [74, 75].

The usefulness of several biomarkers suggested for evaluating/monitoring the severity of the disease might be less evident and their use needs a careful evaluation in the case of diabetes.

Even though we understand that in such a critical situation some of these requests are not so easy to implement, we believe that the best possible action to prevent a worse outcome is essential in any medical act.

## Data Availability

Not applicable.

## Abbreviations

ACE2: Angiotensin-Converting-Enzyme-2
COVID-19: Corona Virus Disease 2019
DPP4 inhibitors: Dipeptidyl-peptidase 4 inhibitors
GLP-1RA: Glucagon-Like-Peptide-1 Receptor Agonists
ICU: Intensive Care Unit
NT-proBNP: N-Terminal-pro-Brain Natriuretic-Peptide
SARS-CoV-2: Severe acute respiratory syndrome coronavirus 2
SGLT-2 inhibitors: Sodium-glucose-transporter-2 inhibitors

## References

[1] Guan WJ, Ni ZY, Hu Y, Liang WH, Ou CQ, He JX, China Medical Treatment Expert Group for Covid-19) (2020). Clinical characteristics of coronavirus disease 2019 in China. N Engl J Med.

[2] Gentile S, Strollo F, Ceriello A (2020). COVID-19 infection in Italian people with diabetes: lessons learned from our future (an experience to be used). Diabetes Res Clin Pract.

[3] Goyal P, Choi JJ, Pinheiro LC, Schenck EJ, Chen R, Jabri A (2020). Clinical characteristics of Covid-19 in New York City. N Engl J Med.

[4] Yan Y, Yang Y, Wang F, Ren H, Zhang S, Shi X (2020). Clinical characteristics and outcomes of patients with severe covid-19 with diabetes. BMJ Open Diabetes Res Care.

[5] Sardu C, Gargiulo G, Esposito G, Paolisso G, Marfella R (2020). Impact of diabetes mellitus on clinical outcomes in patients affected by Covid-19. Cardiovasc Diabetol.

[6] Chase G, Desaive T, Bohe J, Cnop M, De Block C, Gunst J (2018). Improving glycemic control in critically ill patients: personalized care to mimic the endocrine pancreas. Crit Care.

[7] Stoudt K, Chawla S (2019). Don’t sugar coat it: glycemic control in the intensive care unit. J Intensive Care Med.

[8] Guo W, Li M, Dong Y, Zhou H, Zhang Z, Tian C (2020). Diabetes is a risk factor for the progression and prognosis of COVID-19. Diabetes Metab Res Rev.

[9] Zhu L, She ZG, Cheng X, Qin JJ, Zhang XJ, Cai J (2020). Association of blood glucose control and outcomes in patients with COVID-19 and pre-existing Type 2 diabetes. Cell Metabol.

[10] Iacobellis G, Penaherrera CA, Bermudez LE, Mizrachi EB (2020). Admission hyperglycemia and radiological findings of SARS-COv2 in patients with and without diabetes. Diabetes Res Clin Pract.

[11] Shetty S, Inzucchi SE, Goldberg PA, Cooper D, Siegel MD, Honiden S (2012). Adapting to the new consensus guidelines for managing hyperglycemia during critical illness: the Updated Yale Insulin Infusion Protocol. Endocr Pract.

[12] Sharif K, Ghadir S, Jakubowicz D, Amital H, Bragazzi NL (2019). Improved outcome of patients with diabetes mellitus with good glycemic control in the Cardiac Intensive Care Unit: a retrospective study. Cardiovasc Diabetol.

[13] Sardu C, D’Onofrio N, Balestrieri ML, Barbieri M, Rizzo MR, Messina V (2020). Outcomes in patients with hyperglycemia affected by COVID-19: can we do more on glycemic control?. Diabetes Care.

[14] Ceriello A, Zarich SW, Testa R (2009). Lowering glucose to prevent adverse cardiovascular outcomes in a critical care setting. J Am Coll Cardiol.

[15] Mehta P, McAuley DF, Brown M, Sanchez E, Tattersall RS, Manson JJ, HLH Across Speciality Collaboration, UK (2020). COVID-19: consider cytokine storm syndromes and immunosuppression. Lancet.

[16] Bellaver P, Schaeffer AF, Dullius DP, Viana MV, Leitão CB, Rech TH (2019). Association of multiple glycemic parameters at intensive care unit admission with mortality and clinical outcomes in critically ill patients. Sci Rep.

[17] Borzì V, Frasson S, Gussoni G, Di Lillo M, Gerloni R, Augello G, Research Department of FADOI (2016). Risk factors for hypoglycemia in patients with type 2 diabetes, hospitalized in internal medicine wards: findings from the FADOI-DIAMOND study. Diabetes Res Clin Pract.

[18] Ceriello A, Novials A, Ortega E, La Sala L, Pujadas G, Testa R (2012). Evidence that hyperglycemia after recovery from hypoglycemia worsens endothelial function and increases oxidative stress and inflammation in healthy control subjects and subjects with type 1 diabetes. Diabetes.

[19] Chao WC, Tseng CH, Wu CL, Shih SJ, Yi CY, Chan MC (2020). Higher glycemic variability within the first day of ICU admission is associated with increased 30-day mortality in ICU patients with sepsis. Ann Intensive Care.

[20] Kulkarni H, Bihari S, Prakash S, Huckson S, Chavan S, Mamtani M (2019). Independent association of glucose variability with hospital mortality in adult intensive care patients: results from the Australia and New Zealand Intensive Care Society Centre for Outcome and Resource Evaluation Binational Registry. Crit Care Explor.

[21] Takahashi H, Iwahashi N, Kirigaya N, Kataoka S, Minamimoto Y, Gohbara M (2018). Glycemic variability determined with a continuous glucose monitoring system can predict prognosis after acute coronary syndrome. Cardiovasc Diabetol.

[22] Todi S, Bhattacharya M (2014). Glycemic variability and outcome in critically ill. Indian J Crit Care Med.

[23] Ceriello A, Monnier L, Owens D (2019). Glycaemic variability in diabetes: clinical and therapeutic implications. Lancet Diabetes Endocrinol.

[24] Wang A, Zhao W, Xu Z, Gu (2019). Timely blood glucose management for the outbreak of 2019 novel coronavirus disease (COVID-19) is urgently needed. Diabetes Res Clin Pract.

[25] Zhou J, Tan J (2020). Diabetes patients with COVID-19 need better blood glucose management in Wuhan, China. Metabolism.

[26] Klonoff DC (2011). Intensive insulin therapy in critically ill hospitalized patients: making it safe and effective. J Diabetes Sci Technol.

[27] Knopp JL, Chase JG (2020). Clinical recommendations for managing the impact of insulin adsorptive loss in hospital and diabetes care. J Diabetes Sci Technol.

[28] Salinas PD, Mendez CE (2019). Glucose management technologies for the critically ill. J Diabetes Sci Technol.

[29] Fernández Cruz A, Ruiz-Antorán B, Muñoz Gómez A, Sancho López A, Mills Sánchez P, Centeno Soto GA, Puerta de Hierro COVID-19 Study Group, et al.. Impact of glucocorticoid treatment in SARS-COV-2 infection mortality: a retrospective controlled cohort study. Antimicrob Agents Chemother. 2020:AAC.01168-20. 10.1128/AAC.01168-20 PMC7449182 32571831

[30] Berton AM, Prencipe N, Giordano R, Ghigo E, Grottoli S (2020). Systemic steroids in patients with COVID-19: pros and contras, an endocrinological point of view. J Endocrinol Invest.

[31] Prattichizzo F, La Sala L, Rydén L, Marx N, Ferrini M, Valensi P (2019). Glucose-lowering therapies in patients with type 2 diabetes and cardiovascular diseases. Eur J Prev Cardiol.

[32] Ceriello A, Stoian AP, Rizzo M (2020). COVID-19 and diabetes management: what should be considered?. Diabetes Res Clin Pract.

[33] Mehra MR, Ruschitzka F, Patel AN (2020). Retraction-Hydroxychloroquine or chloroquine with or without a macrolide for treatment of COVID-19: a multinational registry analysis. Lancet.

[34] Colson P, Rolain JM, Lagier JC, Brouqui P, Raoult D (2020). Chloroquine and hydroxychloroquine as available weapons to fight COVID-19. Int J Antimicrob Agents.

[35] Quatraro A, Consoli G, Magno M, Caretta F, Nardozza A, Ceriello A (1990). Hydroxychloroquine in decompensated, treatment-refractory noninsulin-dependent diabetes mellitus. A new job for an old drug?. Ann Intern Med.

[36] Marfella R, Paolisso P, Sardu C, Bergamaschi L, D’Angelo EC, Barbieri M (2020). Negative impact of hyperglycaemia on tocilizumab therapy in Covid-19 patients. Diabetes Metab.

[37] Alhazzani W, Møller MH, Arabi YM, Loeb M, Ng Gong M, Fan E (2020). Surviving Sepsis Campaign: guidelines on the management of critically ill adults with coronavirus disease 2019 (COVID-19). Intensive Care Med.

[38] Uijtendaal EV, Zwart-van Rijkom JEF, de Lange DV, Arief Lalmohamed AF, van Solinge WW, Egberts TCG (2015). Influence of a strict glucose protocol on serum potassium and glucose concentrations and their association with mortality in intensive care patients. Crit Care.

[39] Nilsson PM, Cederholm J, Zethelius BR, Eliasson BR, Eeg-Olofsson K, Gudbj Rnsdottir S (2011). Trends in blood pressure control in patients with type 2 diabetes: data from the Swedish National Diabetes Register (NDR). Blood Press.

[40] Pal R, Bhansali A (2020). COVID-19, diabetes mellitus and&nbsp;ACE2: the conundrum. Diabetes Res Clin Pract.

[41] Reynolds HR, Adhikari S, Pulgarin C, Troxel AB, Iturrate E, Johnson SB (2020). Renin–angiotensin–aldosterone system inhibitors and risk of Covid-19. N Engl J Med.

[42] Zhang P, Zhu L, Cai J, Lei F, Qin JJ, Xie J (2020). Association of inpatient use of angiotensin converting enzyme inhibitors and angiotensin II receptor blockers with mortality among patients with hypertension hospitalized with COVID-19. Circ Res.

[43] Cariou B, Hadjadj S, Wargny M, Pichelin M, Al-Salameh A, Allix I, Amadou C (2020). Phenotypic characteristics and prognosis of inpatients with COVID-19 and diabetes: the CORONADO study. Diabetologia.

[44] Guo T, Fan Y, Chen M, Wu X, Zhang L, He T (2019). Cardiovascular implications of fatal outcomes of patients with coronavirus disease 2019 (COVID-19). JAMA Cardiol.

[45] Valensi P, Pariès J, Brulport-Cerisier V, Torremocha F, Sachs RN, Vanzetto G (2005). Predictive value of silent myocardial ischemia for cardiac events in diabetic patients: influence of age in a French multicenter study. Diabetes Care.

[46] Valensi P, Meune C (2019). Congestive heart failure caused by silent ischemia and silent myocardial infarction: diagnostic challenge in type 2 diabetes. Herz.

[47] Valensi PE, Johnson NB, Maison-Blanche P, Extramania F, Motte G, Coumel P (2002). Influence of cardiac autonomic neuropathy on heart rate dependence of ventricular repolarization in diabetic patients. Diabetes Care.

[48] Ziegler D, Zentai CP, Perz S, Rathmann W, Haastert B, Döring A, KORA Study Group (2008). Prediction of mortality using measures of cardiac autonomic dysfunction in the diabetic and nondiabetic population: the MONICA/KORA Augsburg Cohort Study. Diabetes Care.

[49] Liu PP, Blet A, Smyth D, Li H (2020). The science underlying COVID-19: implications for the cardiovascular system. Circulation.

[50] https://www.escardio.org/Education/COVID-19-and-Cardiology/ESC-COVID-19-Guidance. Accessed 24 May 2020.

[51] Cosson E, Nguyen MT, Pham I, Pontet M, Nitenberg A, Valensi P (2009). N-terminal pro-B-type natriuretic peptide: an independent marker for coronary artery disease in asymptomatic diabetic patients. Diabet Med.

[52] Giorda CB, Cioffi G, de Simone G, Di Lenarda A, Faggiano P, Latini R, DYDA Investigators (2011). Predictors of early-stage left ventricular dysfunction in type 2 diabetes: results of DYDA study. Eur J Cardiovasc Prev Rehabil.

[53] Tang O, Daya N, Matsushita K, Coresh J, Sharrett AR, Hoogeveen R (2020). Performance of high-sensitivity cardiac troponin assays to reflect comorbidity burden and improve mortality risk stratification in older adults with diabetes. Diabetes Care.

[54] Preiss D, Sattar N (2016). Research digest: cardiac biomarkers for risk prediction. Lancet Diabetes Endocrinol.

[55] Connors JM, Levy JH (2020). COVID-19 and its implications for thrombosis and anticoagulation. Blood.

[56] Bikdeli B, Madhavan MV, Jimenez D, Chuich T, Dreyfus I, Driggin E (2020). COVID-19 and thrombotic or thromboembolic disease: implications for prevention, antithrombotic therapy, and follow-up. J Am Coll Cardiol.

[57] Ceriello A, Taboga C, Giacomello R, Falleti E, De Stasio G, Motz E, Lizzio S, Gonano F, Bartoli E (1994). Fibrinogen plasma levels as a marker of thrombin activation in diabetes. Diabetes.

[58] Zakai NA, McClure LA, Judd SE, Kissela B, Howard G, Safford M (2017). D-dimer and the risk of stroke and coronary heart disease. The REasons for Geographic and Racial Differences in Stroke (REGARDS) Study. Thromb Haemost.

[59] Ceriello A (1993). Coagulation activation in diabetes mellitus: the role of hyperglycaemia and therapeutic prospects. Diabetologia.

[60] Ceriello A, Giacomello R, Stel G, Motz E, Taboga C, Tonutti L (1995). Hyperglycemia-induced thrombin formation in diabetes The possible role of oxidative stress. Diabetes.

[61] Ceriello A, Marchi E, Palazzni E, Quatraro A, Giugliano D (1990). Low molecular weight heparin restores antithrombin III activity from hyperglycemia induced alterations. Diabetes Metab.

[62] Nusca A, Tuccinardi D, Proscia C, Melfi R, Manfrini S, Nicolucci A (2019). Incremental role of glycaemic variability over HbA1c in identifying type 2 diabetic patients with high platelet reactivity undergoing percutaneous coronary intervention. Cardiovasc Diabetol.

[63] Prattichizzo F, De Nigris V, Spiga R, Mancuso E, La Sala L, Antonicelli R (2018). Inflammageing and metaflammation: the yin and yang of type 2 diabetes. Ageing Res Rev.

[64] Ceriello A (2005). Acute hyperglycaemia: a ‘new’ risk factor during myocardial infarction. Eur Heart J.

[65] Ceriello A, Esposito K, Piconi L, Ihnat MA, Thorpe JE, Testa R (2008). Oscillating glucose is more deleterious to endothelial function and oxidative stress than mean glucose in normal and type 2 diabetic patients. Diabetes.

[66] Cosentino F, Grant PJ, Aboyans V, Bailey CJ, Ceriello A, Delgado V (2020). ESC Scientific Document Group: 2019 ESC guidelines on diabetes, pre-diabetes, and cardiovascular diseases developed in collaboration with the EASD. Eur Heart J.

[67] Dashti-Khavidaki S, Khalili H (2020). Considerations for statin therapy in patients with COVID-19. Pharmacotherapy.

[68] Tikoo K, Patel G, Kumar S, Karpe PA, Sanghavi M, Malek V (2015). Tissue specific up regulation of ACE2 in rabbit model of atherosclerosis by atorvastatin: role of epigenetic histone modifications. Biochem Pharmacol.

[69] Makris D, Manoulakas E, Komnos A, Papakrivou E, Tzovaras N, Hovas A, Zintzaras E, Zakynthinos E (2011). Effect of pravastatin on the frequency of ventilator-associated pneumonia and on intensive care unit mortality: open-label, randomized study. Crit Care Med.

[70] Rogers AJ, Guan J, Trtchounian A (2019). Association of elevated plasma interleukin-18 level with increased mortality in a clinical trial of statin treatment for acute respiratory distress syndrome. Crit Care Med.

[71] Schnell O, Cappuccio F, Genovese S, Standl E, Valensi P, Ceriello A (2013). Type 1 diabetes and cardiovascular disease. Cardiovasc Diabetol.

[72] Sechterberger MK, van Steen SC, Boerboom EM, van der Voort PH, Bosman RJ, Hoekstra JB (2017). Higher glucose variability in type 1 than in type 2 diabetes patients admitted to the intensive care unit: a retrospective cohort study. J Crit Care..

[73] Tatti P, Tonolo G, Zanfardino A, Iafusco D (2020). CoVid-19 and Type 1 Diabetes: every cloud has a silver lining. Searching the reason of a lower aggressiveness of the CoronaVirus disease in Type 1 Diabetes. Diabetes Res Clin Pract.

[74] Guzik TJ, Mohiddin SA, Dimarco A, Patel V, Savvatis K, Marelli-Berg FM (2020). COVID-19 and the cardiovascular system: implications for risk assessment, diagnosis, and treatment options. Cardiovasc Res.

[75] Ceriello A, Standl E, Catrinoiu D, Itzhak B, Lalic NM, Rahelic D, Diabetes and Cardiovascular Disease (D&CVD) EASD Study Group (2020). Issues of cardiovascular risk management in people with diabetes in the COVID-19 era. Diabetes Care.

